# Abnormal Rat Cortical Development Induced by Ventricular Injection of rHMGB1 Mimics the Pathophysiology of Human Cortical Dysplasia

**DOI:** 10.3389/fcell.2021.634405

**Published:** 2021-03-04

**Authors:** Xiaolin Yang, Xiaoqing Zhang, Yuanshi Ma, Zhongke Wang, Kaixuan Huang, Guolong Liu, Kaifeng Shen, Gang Zhu, Tingting Wang, Shengqing Lv, Chunqing Zhang, Hui Yang, Shiyong Liu

**Affiliations:** ^1^National Comprehensive Epilepsy Center, Department of Neurosurgery, Second Affiliated Hospital, Army Medical University, Chongqing, China; ^2^Department of Neurosurgery, First People’s Hospital, Zhaotong, China

**Keywords:** cortical dysplasia, high-mobility group box 1, spike wave, innate immune inflammation, epilepsy

## Abstract

Cortical dysplasia (CD) is a common cause of drug-resistant epilepsy. Increasing studies have implicated innate immunity in CD with epilepsy. However, it is unclear whether innate immune factors induce epileptogenic CD. Here, we injected recombinant human high mobility group box 1 (rHMGB1) into embryonic rat ventricles to determine whether rHMGB1 can induce epileptogenic CD with pathophysiological characteristics similar to those of human CD. Compared with controls and 0.1 μg rHMGB1-treated rats, the cortical organization was severely disrupted in the 0.2 μg rHMGB1-treated rats, and microgyria and heterotopia also emerged; additionally, disoriented and deformed neurons were observed in the cortical lesions and heterotopias. Subcortical heterotopia appeared in the white matter and the gray–white junction of the 0.2 μg rHMGB1-treated rats. Moreover, there was decreased number of neurons in layer V–VI and an increased number of astrocytes in layer I and V of the cortical lesions. And the HMGB1 antagonist dexmedetomidine alleviated the changes induced by rHMGB1. Further, we found that TLR4 and NF-κB were increased after rHMGB1 administration. In addition, the excitatory receptors, *N*-methyl-D-aspartate receptor 1 (NR1), 2A (NR2A), and 2B (NR2B) immunoreactivity were increased, and immunoreactivity of excitatory amino acid transporter 1 (EAAT1) and 2 (EAAT2) were reduced in 0.2 μg rHMGB1-treated rats compared with controls. While there were no differences in the glutamic acid decarboxylase 65/67 (GAD65/67) immunoreactivity between the two groups. These results indicate that the excitation of cortical lesions was significantly increased. Furthermore, electroencephalogram (EEG) showed a shorter latency of seizure onset and a higher incidence of status epilepticus in the 0.2 μg rHMGB1-treated rats; the frequency and amplitude of EEG were higher in the treated rats than controls. Intriguingly, spontaneous electrographic seizure discharges were detected in the 0.2 μg rHMGB1-treated rats after 5 months of age, and spike-wave discharges of approximately 8 Hz were the most significantly increased synchronous propagated waves throughout the general brain cortex. Taken together, these findings indicate that rHMGB1 exposure during pregnancy could contribute to the development of epileptogenic CD, which mimicked some pathophysiological characteristics of human CD.

## Introduction

Cortical dysplasia (CD) is a common cause of drug-resistant intractable epilepsy and is detected in 40–50% of children undergoing epileptic zone resection (Severino et al., 2020). However, the pathophysiological mechanism of CD remains unknown. Increasing evidence has shown that innate immune inflammation plays an important role in epileptogenic CD. The inflammatory receptors toll-like receptor (TLR) and receptor for advanced glycation end products (RAGE) are upregulated in CD and contribute to the high epileptogenicity of CD (Zurolo et al., 2011). Our previous study also indicated that viral infection and TLR-mediated responses are involved in epileptic CD (Liu et al., 2014). The above receptors are specifically activated by damage-associated molecular patterns released by impaired tissue (Ravizza et al., 2018). Specifically, high-mobility group box 1 (HMGB1) participates in the generation and amplification of neuroinflammation and promotes the progression of epileptic diseases with or without CD (Ravizza et al., 2018; Wang et al., 2018). However, there is a lack of evidence that HMGB1 and its downstream signals are involved in the pathogenesis that leads to the epileptogenic CD.

High-mobility group box 1, the key molecule linking tissue damage and stress to innate immune inflammation, is a member of the damage-associated molecular pattern molecules and is widely expressed in the nuclei of normal cells (Ravizza et al., 2018). Moreover, HMGB1 is widely present throughout the developing cortex and thalamus in the E14.5 rodent brain (Fang et al., 2012). HMGB1 is restricted to newly generated neurons of the cortical plate, which suggests that HMGB1 plays an essential role in brain development (Fang et al., 2012). HMGB1 has been shown to facilitate the spontaneous proliferation, migration, and differentiation of neural stem cells *in vitro* (Wang et al., 2014). HMGB1 also promoted neural progenitor differentiation in the hippocampus of Alzheimer’s disease model mice (Meneghini et al., 2013). HMGB1 acts as a macrophage-activating factor by binding to advanced glycation end products (RAGE) and contributes to the proliferation, migration, and sprouting of endothelial cells (Schlueter et al., 2005). The above evidence illustrates a tight relationship between HMGB1 and brain development, and abnormal innate immunity is related to CD development. This suggests that prenatally increased HMGB1 levels promote CD development and may be involved in the pathogenesis of epileptogenic CD. In particular, when neural cells are stimulated by hypoxia, infection, or other injury factors, HMGB1 is released from dying and necrotic cells (Vijayakumar et al., 2019).

In this study, we examined the effects of recombinant human HMGB1 (rHMGB1) on the developing brain of fetal rats (E14.5) by studying the neuronal morphology, inflammation, and excitability. We monitored offspring susceptibility to epileptic seizures and spontaneous epileptic discharges and evaluated the characteristics of raw EEG of the cortex and hippocampus of 0.2 μg rHMGB1-treated rats. In addition, we analyzed the behavioral changes of rHMGB1-treated rats. Our results indicated that rHMGB1-induced abnormal cortical development in rat mimicked the pathophysiology of human CD to some degree.

## Materials and Methods

### Intracerebroventricular Injection of rHMGB1 Into Rat Embryos

As described in a previous study, pregnant SD rats carrying pups at 14.5 days of gestation were anesthetized by using pentobarbiturate, and midline laparotomy was performed to expose the uterus (Walantus et al., 2007). Under cold light source, gently hold the embryo with ring forceps, the lambdoidal and sagittal sutures of the embryonic brain were identified carefully. Then, the injection site was determined to be around 2.5 mm before the lambdoidal suture and 1.5 mm lateral to the right of the sagittal suture. Using a glass pipette (diameter 30–40 μm), two microliters of rHMGB1 diluent (0.1 μg, 0.2 μg, or 0.5 μg rHMGB1) was administrated to all fetuses via transuterine injection targeting the right lateral ventricle of the embryonic brain. The abdominal incision was sutured after filling with warm saline. The same volume of saline was injected into control rats following the same procedure. Then, the pregnant rats were allowed to recover in a warm chamber and provided with topical and systemic analgesia.

### Tissue Preprocessing and Nissl Staining

Briefly, pregnant rats were anesthetized at 24, 48, and 72 h following rHMGB1 administration, and midline laparotomy was performed to expose the uterus. Then, the uterus was rapidly opened, and embryonic brains were collected in sterile tubes and stored at −80°C to examine neuroinflammation. The rHMGB1-treated rats were sacrificed at P60 with pentobarbiturate anesthesia. The malformation lesions were identified and collected under a histology microscope and immediately stored at −80°C. At P7, P60, or after the completion of EEG recording, rats were perfused through the left cardiac ventricle with 4% paraformaldehyde at room temperature after isotonic saline perfusion. The brains were postfixed in 4% paraformaldehyde for 48 h and then processed into frozen sections and dried at −20°C. To observe histological abnormalities, the frozen sections were rehydrated with an ethanol gradient, and the brain tissues were stained by Nissl staining.

### Immunostaining

Frozen sections were subjected to immunostaining. Briefly, the sections were rehydrated with an ethanol gradient, treated with 0.3% hydrogen peroxide and 0.3% Triton X-100 for 30 min, and washed three times in PBS. The sections were then incubated at 4°C overnight with primary antibodies diluted in PBS containing 5% normal goat serum. Primary antibodies against rat NR1 (1:400), NR2A (1:200), NR2B (1:400), GAD65/67 (1:100), GLAST/EAAT1 (1:200), GLT1/EAAT2 (1:400) and NeuN (1:500) were purchased from Abcam Company in China. Primary antibodies against rat SMI311 (1:400) and Vimentin (1:400) were purchased from Proteintech Company in China. On the second day, the sections were washed three times with PBS and incubated with the appropriate horseradish peroxidase-conjugated secondary antibodies (Zhong Shan Jin Qiao, Shanghai, China) for 1 h at 37°C. Finally, the sections were washed again with PBS and reacted with 0.02% 3,3-diaminobenzidine tetrahydrochloride (DAB, Zhong Shan Jin Qiao, Shanghai, China). All sections were counterstained with hematoxylin for 30 s, dehydrated and coverslipped. For immunofluorescence staining, primary antibodies against rat Reelin (1:200), CUX1 (1:200), BRN2 (1:200), SATB2 (1:200), CTIP2 (1:200), TBR1 (1:200) and GFAP (1:200) were used, FITC-conjugated and cy3-conjugated antibodies were selected as the secondary antibodies.

### The Evaluation of Nissl and Immunostaining

According to previous methods (Zaidel et al., 1997; Chana et al., 2003), Nissl staining sections of the primary somatosensory area were viewed using an Olympus BX 63 and captured the pictures from layer II to layer VI (400× magnification, resolution 4080 × 3027) due to the paucity of neurons of layer I (*n* = 6, 18 sections of the primary somatosensory area). All images (at least three visions per layer) were analyzed by ImageJ software for Windows (National Institutes of Health, United States). First, the scale was set to micrometers with a reticle, and then the area, shape description and fit ellipse were selected in the “set measurements” menu. The background of the picture was subtracted from the 2D rolling ball. Overlapping neurons were watershed, and glia were recognized by their shape, size and dark staining. Finally, a threshold was enabled, and a wand tool was used to select each neuron for measurements. Neurons on the edge and indistinguishable overlapping neurons were excluded. Using the same method, the Reelin, CUX1, BRN2, SATB2, CTIP2, TBR1, and GFAP immunofluorescence staining was used for neuron and astrocyte counting in every layer of cortical lesions. Then the result was averaged to 0.1 mm^2^.

The immunohistochemical sections were reviewed according to a previous method (Jensen, 2013). To measure the intensity of NR1, NR2A, NR2B, GAD65/67, EAAT1, and EAAT2 immunoreactivity, we selected three sections of cortical lesions (mainly including somatosensory cortex) in rHMGB1-treated rats and the corresponding cortex from controls (*n* = 5 rats in each group). Then we captured three visions (100× magnification, resolution 4080 × 3027) in each section. The quantitative analyses of visions were performed using ImageJ software. The software can extract the DAB and hematoxylin image by the IHC profiler plugin, respectively. First, we extract the DAB image, transformed the DAB image into 8-bit grayscale images, and selected the uncalibrated OD. Second, the staining density of normal subcortical white matter was subtracted for standardization. Third, the “integrated density,” “area,” and “limit to threshold” were selected from the “set measurements” menu. Then, we selected the proper threshold and measured the staining density of each cell body. Finally, the staining density values were averaged to 0.1 mm^2^. The hippocampus of rats from each group was also analyzed using the same methods.

The SMI311-positive pyramidal neurons were evaluated using somatic area and apical dendrite thickness. According to the previous study (Colciaghi et al., 2011): (1) at least 100 neurons were selected; (2) no nucleolus on the plane of the section was excluded; (3) the cell areas and diameters and apical dendritic thickness were measured at 5–15 μm from the upper edge of the nucleus. Besides, dysmorphic neurons were identified by neuronal orientation, size, cytoskeletal structure, and atypical dendrite, as described in previous study (Palmini et al., 2004).

Although the above analysis has limitations compared with the stereological method, any counting bias should equally affect all samples considered (Colciaghi et al., 2011).

### Western Blot

Embryonic rat brains were homogenized with RIPA buffer containing 10% protease inhibitor (Beyotime, Shanghai, China). The tissue lysate was centrifuged at 4°C (12000 rpm) to extract the protein supernatant. The protein concentration was examined by a Protein Quantitation Kit (Abcam, Cambridge, United Kingdom) according to the manufacturer’s instructions. Protein (50 μg/lane) was isolated by 10% SDS–polyacrylamide gel electrophoresis and then transferred onto polyvinylidene fluoride membranes (Millipore, Temecula, CA, United States) in glycine transfer buffer. The polyvinylidene fluoride membranes were blocked in milk containing 5% non-fat-dried power for 2 h and incubated overnight at 4°C with primary antibodies against rat NF-κB (1:800; Proteintech, China), TLR4 (1:600; Abcam, United Kingdom), and GAPDH (1:1000; CST, Beverly, MA, United States). The next day, the membranes were washed three times in PBS and incubated with a horseradish peroxidase-conjugated anti-rabbit or anti-mouse secondary antibody (1:1000; Zhong Shan Jin Qiao, China) for 1 h at 37°C. The cortical lesions homogenate of adult rats (P60) was examined following the above methods. The primary antibodies against rat were NR2A (1:400; Abcam, United Kingdom), NR2B (1:400; Abcam, United Kingdom), NR1 (1:400; Abcam, United Kingdom), EAAT1 (1:400; Abcam, United Kingdom), and anti-rat EAAT2 (1:400; Abcam, United Kingdom). All immunoreactivity (IR) bands were directly viewed by chemiluminescence and analyzed by ImageJ. The optical density was calculated relative to the optical density of GAPDH.

### The Antagonism of rHMGB1-Induced Changes

Dexmedetomidine (Dex) is a selective α2 receptor agonist and effectively inhibits TLR4/NF-κB signal pathway. Therefore, we further studied the effect of dexmedetomidine on the 0.2 μg rHMGB1 treated rats. Three rats carried pups were divided into control, rHMGB1-treated, and rHMGB1 + Dex group randomly. The control group only were injected saline into the embryonic lateral ventricle. In the rHMGB1-treated group, 0.2 μg rHMGB1 were administrated to the right lateral ventricle of the embryonic brain. According to previous studies (Gu et al., 2011; Rong et al., 2017; Cheng et al., 2018), 10 μg/kg Dex was injected subcutaneously into pregnant rats awakened from anesthesia after 0.2 μg rHMGB1 delivery into the embryonic brain. The cerebral cortical changes of offspring were observed using Nissl staining in postnatal days 7.

### Placement of Electrodes

Recording electrodes for EEG monitoring were placed on the bilateral frontal cortices, hippocampus, and occipital cortices of the rats. After sedation with pentobarbital, two silver electrodes were inserted into the bilateral hippocampus (anteroposterior, −4.7 mm; lateral, ±5.0 mm; 5.5 mm depth from the surface), and the screws were implanted on bilateral frontal cortices (anteroposterior, 3.0 mm; lateral, ±2.5 mm) and bilateral occipital cortices (anteroposterior, −7.5 mm; lateral, ±4.0 mm), while the reference and ground electrodes were positioned on the olfactory bulb and cerebellum, respectively. All rats were allowed to recover in a warm chamber. Then, the rats were singly housed in a dedicated cage and provided with topical and systemic analgesia and antibiotics.

### Seizure Behavior Evaluation

Rats received pilocarpine (0.7 mg/5 μl) via intracerebroventricular injection and were observed to assess the latency of onset seizure and status epilepticus. The onset of status epilepticus was defined by the time between pilocarpine injection and continuous seizure activity (Stages 4 or 5) (Colciaghi et al., 2011). Seizure scores were evaluated during the next 2 h. Behavioral seizure score: stage 1: akinesia, staring and salivation; stage 2: head clonus, forelimb clonus, and wet dog shakes; stage 3: rearing with forelimb clonus; stage 4: rearing and falling with generalized convulsions and status epilepticus; stage 5: death. After 3 h of status epilepticus, all surviving rats received intraperitoneal diazepam to stop the seizure. Then, the rats were put into a warm chamber to recover and were administered saline intraperitoneally.

### Behavioral Tests

All behavioral tests were performed in 3-month-old rats (*n* = 20). The laboratory technician was blinded to all experiments. After the tests, all rats were allowed to rest for 24 h. Open-field tests, novel object recognition tests, and Morris water maze tests were performed. The following are brief descriptions of the test methods. Open-field test: rats were placed in the center of an arena (60 × 60 × 60 cm) and recorded for 10 min using an open-field apparatus (Biowill, Shanghai, China). The total distance, average speed, center active time, and distance and periphery active times were recorded. Novel object recognition test: before testing, rats were put into the empty testing apparatus for 5 min. During the training, two identical objects were positioned in the right and left corners; the rats were allowed to freely explore both objects for 5 min. After the completion of training, the rats were tested for object memory. After the left top corner object was replaced with a novel object that was a similar size but a different shape and color than both previous objects, the rats were put into the center and allowed to explore the objects for 5 min. The time spent exploring the novel and familiar objects were recorded and analyzed. Morris water maze test: this test was performed as described in previous studies. Rats were trained to find a hidden platform in the third quadrant of the pool according to a distal visual cue. The rats were trained for six consecutive days at the same time (9:00 PM) each day with four quadrant starting points. On day 7, the probe tests were carried out after the platform was removed.

### EEG Acquisition and Analysis

Two weeks after surgery, the rHMGB1-treated rats were divided into two groups (A, *n* = 10 and B, *n* = 10). The EEGs of the control group and group A rHMGB1-treated rats (*n* = 10 rats) were monitored after pilocarpine administration 3 months after birth. Spontaneous discharges and behavioral seizures were monitored for 3 months for 24 h every day in the B group (*n* = 10 rats). The recording settings were a low-frequency filter of 0.1 Hz, a high-frequency filter of 500 Hz, and samples of 3000 Hz. The intensity and frequency distribution of EEG was obtained from the power spectrum by fast Fourier transform with a 2-s Hanning window across a continuous 1–500 Hz frequency range. The absolute power (μV^2^) of each EEG frequency band [delta (1–4 Hz), theta (4–8 Hz), alpha (8–13 Hz), beta (13–30 Hz), lower gamma (30–60 Hz), higher gamma (60–80 Hz), ripple (80–250 Hz) and fast ripple (250–500 Hz)] was compared between the rHMGB1-treated rats and the control rats at selected pre-pilocarpine resting periods (background activity) and post-pilocarpine ictal periods. Time–frequency and wavelet analyses were performed using Morlet wavelets in Brainstorm, which was developed with MATLAB software. The synchrony of spontaneous spike-wave discharges was observed through coherence and cross-correlation analyses by EEGLAB and custom codes.

### Statistical Analysis

The proper sample sizes were estimated on the previous model and seizure experiments. G^∗^Power 3 was used to calculate the sample size (Faul et al., 2007). For each experiment process, the sample size was estimated for an effect size of 50 to 70% using SD calculated from the control population and a power at 0.8 (β = 0.2) and an α of 0.05. The sample of each experiment was *n* ≥ 3. For example, in Figure 6C (the NR2A protein levels of cortical lesions homogenate), using unpaired *t*-test (two groups, two-tails), six mice in each group would be sufficient to reach significance with a power of 0.9 and an α of 0.05 for the presented data. The schematic diagram including methods and number of animals in the different experimental procedures were shown in Supplementary Figure 1.

The data are presented as the mean ± SEM and were plotted by GraphPad 7.0 software. The neuronal shape and angle were analyzed by one-way ANOVA. The statistical analysis of the numbers of neurons and astrocytes from every layer was performed by using two-way ANOVA. Western blots and immunohistochemistry results were analyzed by two-way ANOVA or unpaired *t*-test. The latency of pilocarpine-induced seizures was analyzed using unpaired *t*-tests. The rate of status epilepsy and mortality were compared with the chi-square test. We compared the powers of different frequency bands between the control and rHMGB1-treated groups using two-way ANOVA. Furthermore, the powers of different frequency bands of the spontaneous epileptic discharges were also analyzed by two-way ANOVA.

## Results

### General Information

None of the control pups or 0.1 μg rHMGB1-injected pups were born dead, 23% of the 0.2 μg rHMGB1-injected pups were born dead, and all of the 0.5 μg rHMGB1-injected pups died. There were no differences in the diet, daily activities, or growth processes, including fur, body weight, and brain weight, between the rHMGB1-treated groups and controls. Animals survived until postnatal days 7, days 60, or the completion of EEG recording.

### Abnormalities in Brain Structure

**FIGURE 1:**
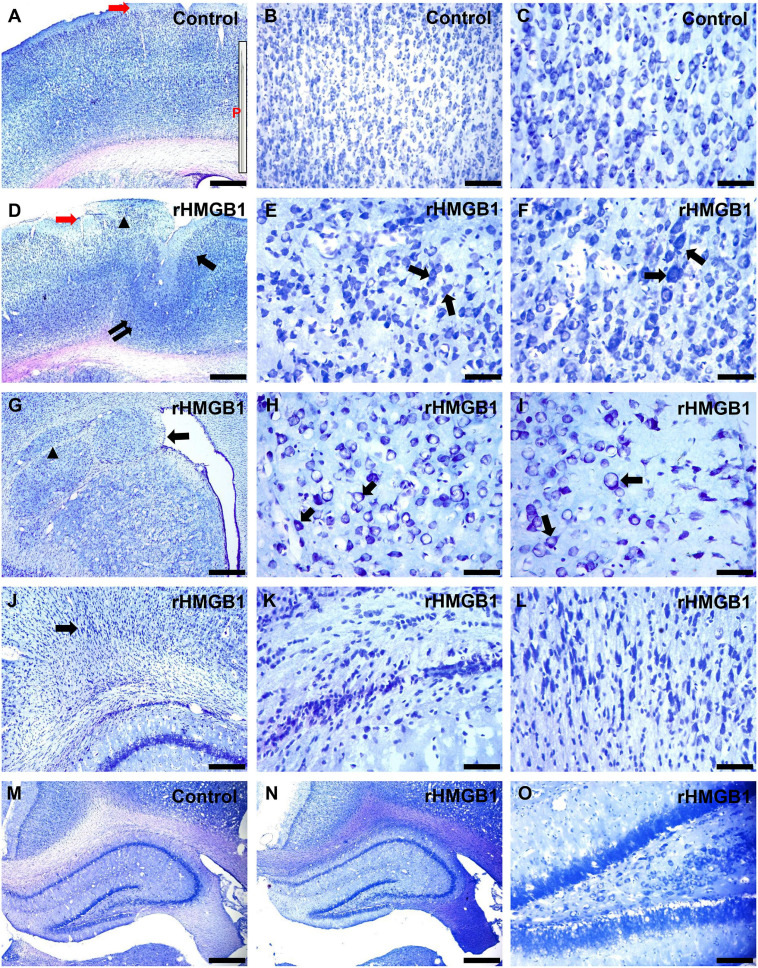
Morphological effects of 0.2 μg rHMGB1 treatment on cortical and hippocampal tissues. **(A–C)** Nissl staining showed clear cortical layers and neural orientation in saline-injected controls (primary somatosensory area, –0.84 mm from bregma), the inset (P) is the needle (30–40 μm), red arrow is the injection site. **(D–F)** Focal cortical layer disorganization, heterotopic cortex, and microgyria (**D**, arrows), including disorganized neurons and disoriented neurons (**E,F**, arrows) in the primary somatosensory area (–0.84 mm from bregma) of rHMGB1-treated versus control rats. Panels **(G–I)** show the paraventricular nodules (**G**, arrow, +1.2 mm from bregma), including disoriented neurons (**H,I**, arrows) and subcortical heterotopia (**G**, triangle). **(J–L)** Delayed nerve cell migration and retention in the gray-white junction (**J**, arrow) in rHMGB1-treated rats (–3.36 mm from bregma). **(M–O)** There are no significant differences in the general morphology of the hippocampus (–3.0 mm from bregma) between controls and rHMGB1-treated rats. rHMGB1, rHMGB1-treated. Scale bars, 500 μm for panels **(A,D,G,J,M,N)**; 100 μm for panels **(B,O)**; 50 μm for the remaining images.

In this study, Nissl staining was performed and showed that the cortical lesions were mainly located in the right primary somatosensory area (S1) (*n* = 6, around +1.56 mm to −2.4 mm from bregma; Mean width, 1.8 ± 0.6 mm). Well-preserved cortical lamination, neuronal size, shape, and orientation were observed in the cortex of the control rats (Figures 1A–C and Supplementary Figure 2). However, in the group treated with 0.2 μg rHMGB1, cortical lamination was severely disrupted (Figure 1D, arrow), and cortical lesions (Figure 1D, double arrow) and heterotopia (Figure 1D, triangle) were observed; furthermore, neuronal morphology and size were variable (Figures 1E,F,H,I, arrows and Supplementary Figures 2A–J); the proportion of large size neurons (180–240 μm^2^) were increased in cortical layers II and III (Supplementary Figures 2A,B), while the proportion of small size neurons (60–80 μm^2^) were increased in cortical layers IV, V and VI of the 0.2 μg rHMGB1-treated rats (Supplementary Figures 2C–E) versus controls. There were different shapes of neurons in cortical layers II–VI of the 0.2 μg rHMGB1-treated rats compared with controls (Supplementary Figures 2F–J), and only the neurons of layers IV and V displayed the abnormal orientation (Supplementary Figures 2K–O). Heterotopic nodules consisting of disoriented neurons were found in the periventricular region (Figures 1G–I). Subcortical heterotopia was found in the white matter (Figure 1G) and the gray–white junction of 0.2 μg rHMGB1-treated rats (Figures 1J–L). However, no abnormal shape, size, and orientation neurons were observed in the cortical layers II–VI of controls and 0.1 μg rHMGB1-treated rats (Supplementary Figures 2A–O); there were no significant differences in the hippocampus’s general morphology between controls and rHMGB1-treated rats (Figures 1M–O).

**FIGURE 2:**
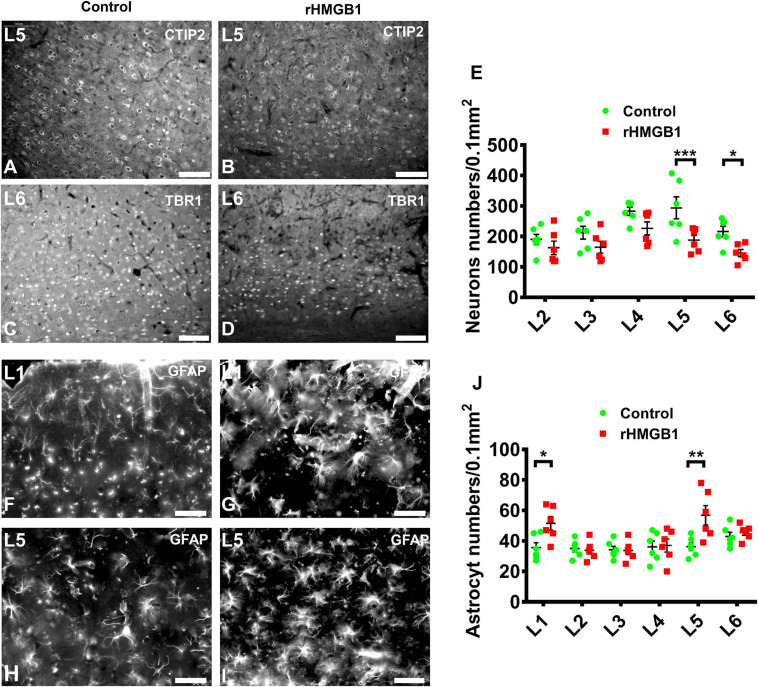
The immunofluorescence analysis of neuron and astrocyte in cortical layer I–V. **(A–D)** Representative photographs of cortical layer V and VI obtained from S1 (+1.08 mm from bregma) of control **(A,C)** and rHMGB1-treated rats **(B,D)**. The CTIP2 **(A,B)** and TBR1 **(C,D)** positive cells were significantly decreased in cortical layer V and VI of rHMGB1-treated rats versus controls (**E**, ***P = 0.0005 and *P = 0.0236, respectively); the number of NeuN positive cells in other cortical layer were not different between rHMGB1-treated rats and controls. **(F–I)** The representative photographs of cortical layer I and VI obtained from S1 (+1.32 mm from bregma) of rHMGB1 **(G,I)** and control **(F,H)**. The GFAP positive astrocyte were increased in layer I and V of rHMGB1-treated rats versus controls (**J**, **P = 0.0194 and **P = 0.0016, respectively); the number of GFAP positive cells in other cortical layer were not different between rHMGB1 and control rats. Data are presented as mean ± SEM (*n* = 6 rats from each group). S1, primary somatosensory area; rHMGB1, rHMGB1-treated. L1–L5, layer I–V; Scale bars, 100 μm for panels **(A–E)**; 50 μm for panels **(F–J)**.

**FIGURE 3:**
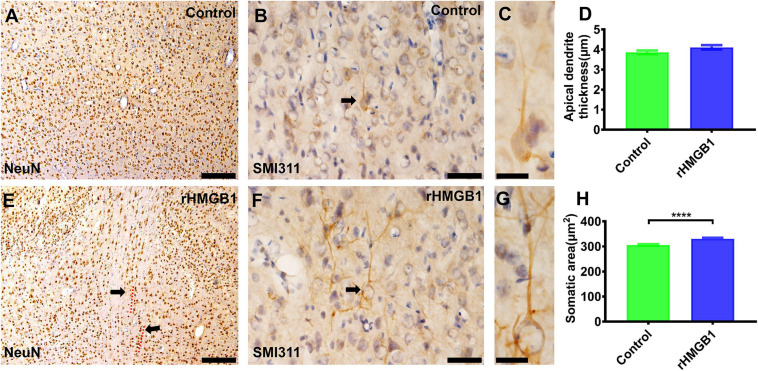
The immunohistochemical staining of NeuN and SMI311 in cortical lesions. **(A,E)** There were linearly aligned neurons in the cortical lesions (**E**, dotted curve and arrows) in rHMGB1-treated rats (–2.4 mm from bregma). **(B,F)** The stronger SMI311-positive neurons in cortical lesions compared with the control cortex (**B,F**, arrows, cortical layer III) (S1, –2.64 mm from bregma) and **(H)** the somatic size of deformed neurons were bigger than the neurons in control rats (****P < 0.0001), the apical dendrite thickness were increased but not significant different in rHMGB1-treated rats versus controls **(D)**. Data are presented as mean ± SEM. S1, primary somatosensory area; rHMGB1, rHMGB1-treated. Scale bar, 200 μm for panels **(A,E)**; 50 μm for panels **(B,F)**. 20 μm for panels **(C,G)**.

The specific cortical layer marker, including Reelin, CUX1, BRN2, SATB2, CTIP2, and TBR1, was used to represent neurons in layer I–VI, respectively. CTIP2 and TBR1 immunofluorescence revealed that the number of neurons was decreased in layers V and VI of the cortical lesions (right S1) compared with controls (Figures 2A–E; *n* = 6, *P* = 0.0005 and *P* = 0.0236, respectively), there no significant difference in other layer neurons of rHMGB1-treated rats versus controls (Figure 2E). Interestingly, NeuN immunostaining showed linearly aligned neurons in the cortical lesions (Figure 3E, dotted curve and arrows) compared to controls (Figure 3A), which is similar to the microcolumn in the pathology of human FCDIa. SMI311 staining was used to display the dysmorphic neurons in the cortical lesions. The results showed the neurons of stronger SMI311 immunoreactivity were observed in the cortical lesions of HMGB1-treated rats than controls (Figures 3B,C,F,G). The somatic size of SMI311-positive neurons was bigger than neurons in control rats (Figures 3C,G,H, *P* < 0.0001), which were called deformed neurons. The apical dendrite thickness of deformed neurons was increased, though no statistical difference (Figure 3D). However, balloon cells were not found in the cortical lesions using Vimentin staining. Glial fibrillary acidic protein (GFAP)-positive astrocytes displayed significantly increased cell density in layers I and V of the cortical lesions (Figures 2F–J, *n* = 6, *P* = 0.0194 and *P* = 0.0016, respectively).

### Neuroinflammation in 0.2 μg rHMGB1-Treated Rats

**FIGURE 4:**
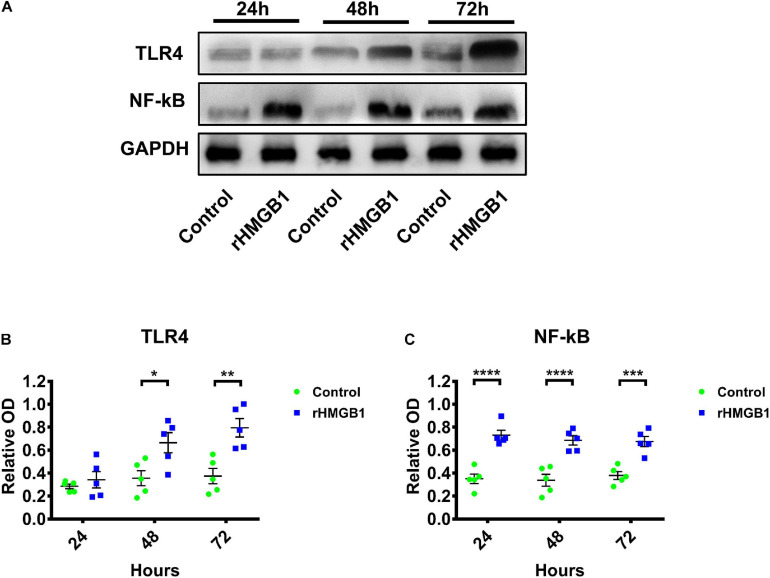
The western blot of TLR4 and NF-κB in rHMGB1-treated and control cortex from three time points. **(A)** The represent band of TLR4 and NF-κB. **(B)** Statistical analysis showing TLR4 were upregulated in rHMGB1-treated rats compared with controls (*P* = 0.8761 at 24 h, *P = 0.0156 at 48 h, **P = 0.0026 at 72 h); **(C)** NF-κB were also significant increased (****P < 0.0001 at 24 h, ****P < 0.0001 at 48 h, ***P = 0.0002 at 72 h) in rHMGB1-treated rats versus controls. Data are presented as mean ± SEM (*n* = 5 rats from each group). rHMGB1, rHMGB1-treated; TLR4, toll like receptor 4; NF-κB, NF-kappa B.

We detected neuroinflammation in embryonic rat brains at 24, 48, and 72 h after injected with 0.2 μg rHMGB1. The results showed that the expression of TLR4 (Figures 4A,B; *n* = 5; *P* = 0.8761 at 24 h, *P* = 0.0156 at 48 h, *P* = 0.0026 at 72 h) and NF-κB were upregulated in the 0.2 μg rHMGB1-treated rats compared with that in the controls (Figures 4A,C; *n* = 5; *P* < 0.0001 at 24 h, *P* < 0.0001 at 48 h, *P* = 0.0002 at 72 h).

### Antagonism of Dexmedetomidine on Change Induced by rHMGB1

**FIGURE 5:**
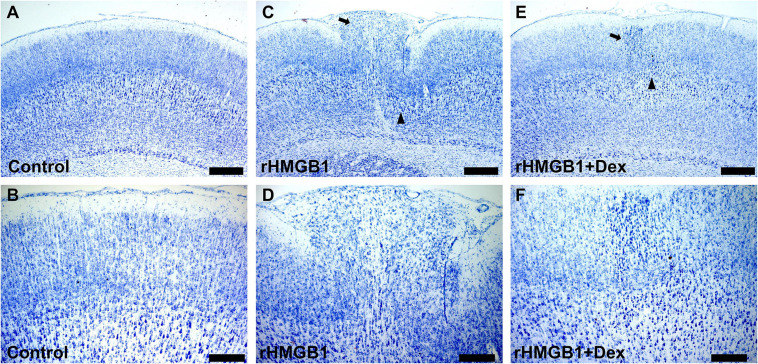
The antagonistic effect of dexmedetomidine on rHMGB1. **(A,B)** Representative photographs of the primary somatosensory area (+0.6 mm from bregma) from controls. **(C,D)** Representative photographs of the primary somatosensory area (+0.6 mm from bregma) from rHMGB1-treated rats; cortical lesions (**C**, triangle) and heterotopia (**C**, arrow) in rHMGB1-treated rats (cortical lesions, 2.54 ± 0.45 mm^2^; heterotopia, 0.60 ± 0.12 mm^2^). **(E,F)** Representative photographs of the primary somatosensory area (+0.48 mm from bregma) from rHMGB1 + Dex rats; cortical lesions and heterotopia were also observed (**E**, triangle and arrow; cortical lesions, 1.74 ± 0.22 mm^2^; heterotopia, 0.22 ± 0.06 mm^2^). Data are presented as mean ± SEM (*n* = 5 rats from each group). rHMGB1, rHMGB1-treated; Dex, dexmedetomidine. Scale bar 500 μm for **(A,C,E)**: 200 μm for **(B,D,F)**.

Dexmedetomidine (Dex) is a selective α2 receptor agonist and effectively inhibits TLR4/NF-κB signal pathway. Therefore, we further studied the effect of dexmedetomidine on the 0.2 μg rHMGB1 treated rats. The results showed the cortical malformation (Figures 5C,D) and heterotopia (Figure 5C) were observed in rHMGB1-treated rats (*n* = 5, P7 days; cortical lesions, 2.54 ± 0.45 mm^2^; heterotopia, 0.60 ± 0.12 mm^2^) compared to controls (Figures 5A,B) and were larger than that in rHMGB1 + Dex treated rats (Figures 5E,F, cortical lesions, 1.74 ± 0.22 mm^2^; heterotopia, 0.22 ± 0.06 mm^2^).

### Neuronal Excitability in 0.2 μg rHMGB1-Treated Rats

**FIGURE 6:**
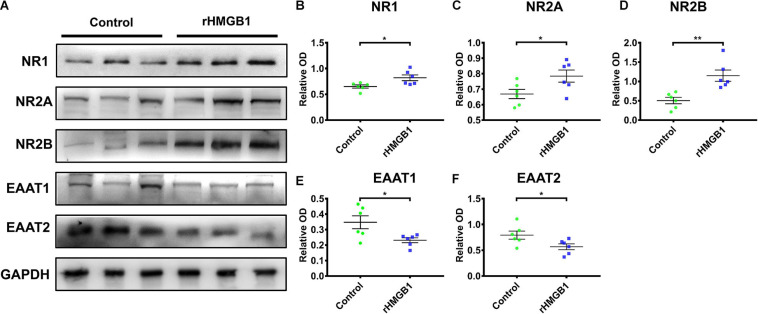
The western blot of cortical lesions and control cortex. **(A)** The represent band of NR1, NR2A, NR2B, EAAT1 and EAAT2. **(B–D)** NR1, NR2A and NR2B protein levels were increased in cortical lesions homogenate of P60 days rHMGB1 rats versus controls (0.65 ± 0.03 versus 0.82 ± 0.06 **P = 0.0227, 0.67 ± 0.03 versus 0.78 ± 0.04 *P = 0.0405, 0.50 ± 0.08 versus 1.15 ± 0.15 **P = 0.0032, respectively); **(E,F)** EAAT1 and EAAT2 were also significant decreased in rHMGB1 group compared with control rats (0.35 ± 0.04 versus 0.23 ± 0.02 *P = 0.0266, 0.79 ± 0.08 versus 0.57 ± 0.06 *P = 0.0421, respectively). Data are presented as mean ± SEM (*n* = 6 rats from each group). rHMGB1, rHMGB1-treated; NR2A, *N*-methyl-D-aspartate receptor 2A; NR2B, *N*-methyl-D-aspartate receptor 2B; NR1, *N*-methyl-D-aspartate receptor 1; EAAT1, excitatory amino acid transporter 1; EAAT2, excitatory amino acid transporter 1.

Western blot analysis showed that the NR1, NR2A, and NR2B protein levels were increased in cortical lesions homogenate of rHMGB1-treated rats (P60 days) compared with controls (Figures 6A–D, *n* = 6; *P* = 0.0227, *P* = 0.0405, *P* = 0.0032, respectively); EAAT1 and EAAT2 were also significantly decreased (Figures 6A,E,F, *n* = 6; *P* = 0.0266, *P* = 0.0421, respectively). The NR2A and NR2B protein levels were not significantly different in the hippocampus between rHMGB1-treated rats and controls (data no shown). Immunohistochemical analysis also indicated that NR1, NR2A, and NR2B immunoreactivity was significantly increased in cortical lesions (Supplementary Figures 3A–I, *n* = 5; *P* = 0.0104, *P* = 0.0253, *P* < 0.0001, respectively) compared with controls. However, the immunoreactivity density of GAD56/67 was not different between control and cortical lesions (Supplementary Figures 3J–L, *n* = 5, *P* = 0.40). EAAT1 and EAAT2 immunoreactivity were decreased in cortical lesions (Supplementary Figures 3M–R, *n* = 5, *P* = 0.0017, *P* = 0.0397, respectively) versus controls. Furthermore, stronger NR2A and NR2B IR were found in only the CA3 of the rHMGB1-treatment group (Supplementary Figures 4A–F, *p* < 0.001 and *p* < 0.0001, respectively).

### Pilocarpine-Evoked Seizures in 0.2 μg rHMGB1-Treated Rats

**FIGURE 7:**
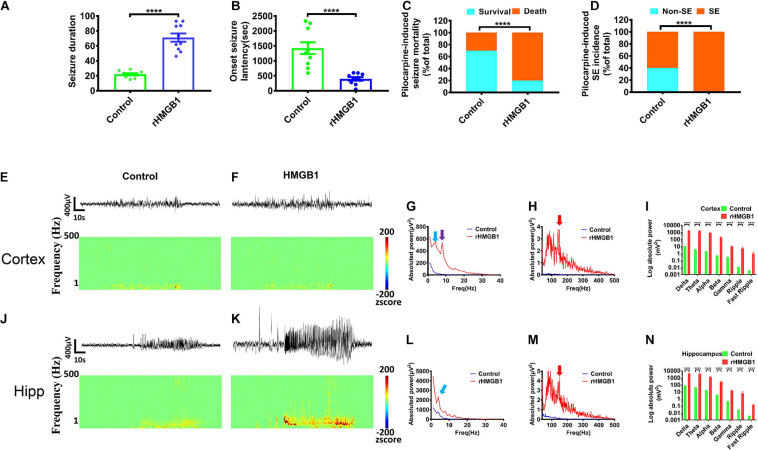
Pilocarpine-induced effects were obtained from rHMGB1-treated rats and controls. **(A,B)** The onset latency of seizures was shorter in rHMGB1-treated rats after pilocarpine administration (*n* = 10, 1423 ± 198.3 versus 398.9 ± 56.55, ****p < 0.0001), and the seizure duration was longer than controls (*n* = 10, 22.03 ± 1.40 versus 71.13 ± 5.61, ****p < 0.0001). **(C,D)** The rate of status epilepticus and mortality were higher in rHMGB1-treated group than controls (****p < 0.0001). **(E–N)** The raw EEG showed the amplitude were significantly increase. The time–frequency analysis displayed all bands power were significantly higher in rHMGB1-treated rats than controls during seizure after pilocarpine injection. Especially, the around 4 Hz and 150 Hz bands power were the most significant in both cortex and hippocampus (**G,L**, blue arrows; **H,M,** red arrows), but only the around 8 Hz peak in cortex (**G**, purple arrows). Data are presented as mean ± SEM. SE, status epilepticus; rHMGB1, rHMGB1-treated; Hipp, hippocampus.

The rHMGB1-treated rats displayed a longer seizure duration than controls (Figure 7A, *n* = 10, *P* < 0.0001) and a shorter latency of seizure onset (Figure 7B, *n* = 10, *P* < 0.0001). The incidences of status epilepticus and mortality were also higher in the rHMGB1-treated group than controls [control, 60% SE (*n* = 6) and 30% (*n* = 3) mortality; rHMGB1-treated group, 100% SE (*n* = 10) and 80% mortality (*n* = 8), *n* = 10, *P* < 0.0001, chi-square test] (Figures 7C,D). The behavioral seizure scores were 3/5 in the control group and 4/5 in the rHMGB1-treated group. Therefore, the severity of the seizures triggered by a single pilocarpine dose was significantly higher in the rHMGB1-treated rats than in the control rats. Electroencephalography showed a high voltage spike, particularly in the hippocampus, before seizure onset after pilocarpine administration. Cortical discharges followed the hippocampal seizures. Quantitative measurement of the power spectra showed that the absolute powers of all bands after pilocarpine administration in the cortex and hippocampus of the rHMGB1-treated rats were increased compared with those of the control rats (Figures 7E–N, *p* < 0.0001), and the powers of approximately 4 Hz, 8 Hz and 150 Hz bands were the most significantly increased in the rHMGB1-treated rats versus controls (Figures 7G,H), but there was no obvious 8 Hz peak in the hippocampus (Figure 7L).

**FIGURE 8:**
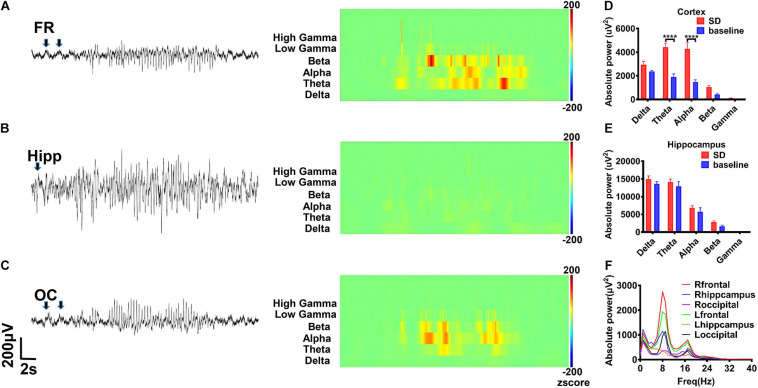
The rHMGB1-treated rats displayed non-convulsive spontaneous spike-wave discharges. **(A–C)** The raw EEG recording and wavelet analysis of non-convulsive spontaneous discharges; the theta, alpha, beta, and gamma power were increased in rHMGB1-treated group and the slow wave was before spontaneous spike-wave discharges (arrows). **(D,E)** The statistics of different bands power demonstrated compare with self-baseline, only the cortical powers of theta and alpha bands were significantly increased (Data are presented as mean ± SEM, ****p < 0.0001). **(F)** The power spectra showed around 8 Hz is the most significant on frontal electrode. *n* = 8 rats from each group. FR, frontal lobe; Hipp, hippocampus; OC, occipital lobe; SD, spontaneous discharges.

In addition, we did not record spontaneous epileptic discharges in the rHMGB1-treated and control rats before 5 months postnatal. Paroxysmal synchronous non-convulsive spontaneous spike-wave discharges (SWDs) were detected in the cortex of the 8/10 rHMGB1-treated rats beyond 5 months postnatal. Moreover, slow waves appeared before the SWDs (Figures 8A–C, arrows); the rats did not show abnormal behavior when the SWDs appeared. The time–frequency analysis showed that the SWDs were concentrated in the theta, alpha, and beta bands, and the power spectrum analysis found that the theta-alpha band (approximately 8 Hz) was the most significantly increased (Figures 8A–D,F, *p* < 0.0001). However, the power of the hippocampus was not different from baseline (Figures 8B–E,F).

**FIGURE 9:**
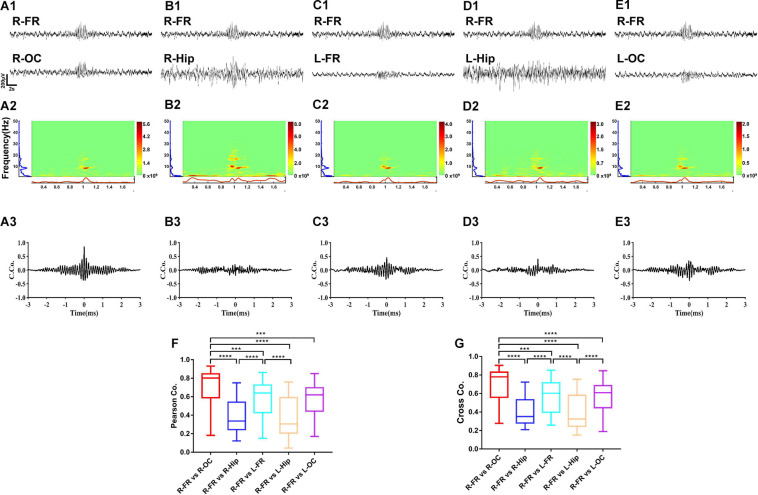
The synchrony degree of spontaneous discharges between different brain regions. The results showed the right frontal cortex and occipital cortex had the highest degree of synchrony **(A1–G)**. Intriguing, synchronization of spontaneous spike-wave discharges was concentrated to 8 Hz **(A2–E2)**. The coherence and cross-correlation analysis also showed right frontal cortex and occipital cortex had the maximum coefficient, secondly the coefficient between right frontal cortex and left frontal cortex **(A3–G)**. Nonetheless, the spontaneous spike-wave discharges between cortex and hippocampus displayed various degree of synchrony. Data are presented as mean ± SEM. *n* = 8 rats from each group, ***p < 0.001, ****p < 0.0001. R-FR, right frontal lobe; L-FR, left frontal lobe; R-Hip, right hippocampus; L-Hip, left hippocampus; R-OC, right occipital lobe; L-OC, left occipital lobe.

Coherence and cross-correlation analyses were employed to examine the temporal and spatial propagation of SWDs. The results indicated that the degree of synchrony was the most significant between the frontal cortex and the ipsilateral occipital cortex (Figures 9A1–G, *p* < 0.0001). Intriguingly, synchronization of spontaneous discharges between the remote cortex areas was concentrated at approximately 8 Hz (Figures 9A2–E2), suggesting that these slow waves may be involved in the generalization and synchronization of epileptic discharges across various brain cortices.

### Behavioral Changes in 0.2 μg rHMGB1-Treated Rats

**FIGURE 10:**
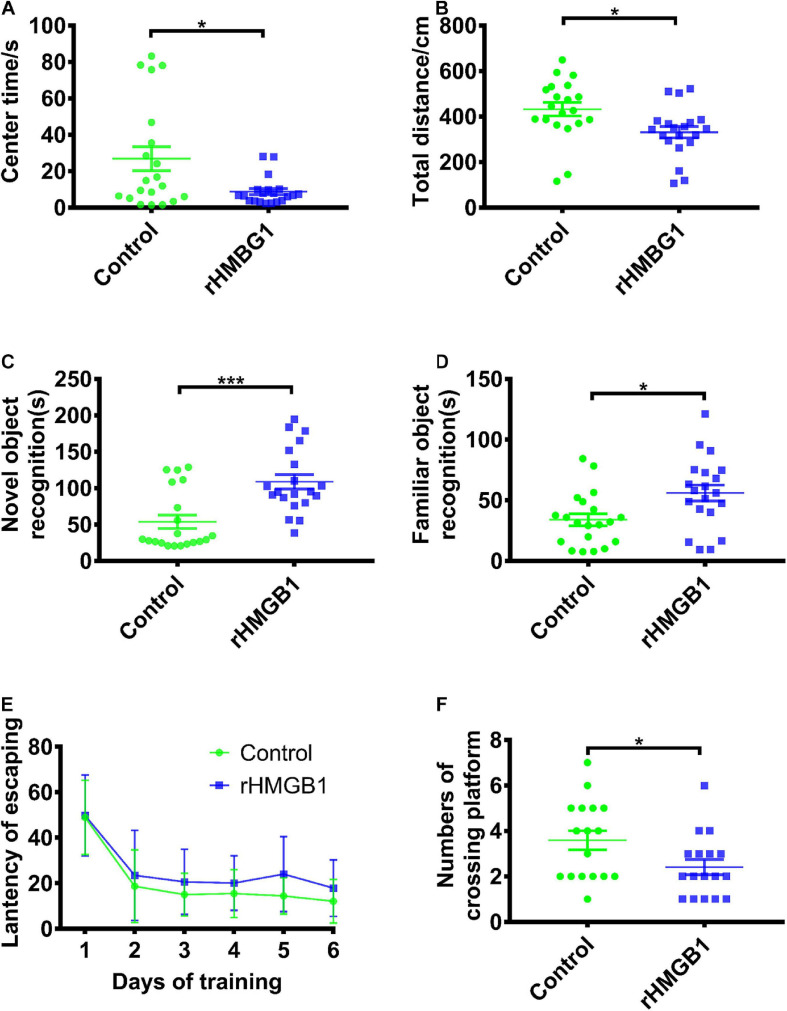
Rats displayed impaired cognition and spatial learning and memory in rHMGB1-treatment group. **(A,B)** The center time and total activity distance. The rHMGB1-treated rats spent less time in the center than the control rats (26.85 ± 6.536 versus 8.757 ± 1.692, *P = 0.0133) and had a decrease in the total distance traveled (432.5 ± 29.73 versus 331.3 ± 25.19, *P = 0.0108). **(C,D)** Average time exploring novel and familiar object during 5 min test. The rHMGB1-treated rats spent more time examining novel and familiar objects than the control rats (53.86 ± 9.223 versus 108.9 ± 9.981 ***P = 0.0002, 34.01 ± 4.9 versus 56.01 ± 6.6 *P = 0.0107, respectively). **(E,F)** The escape latency during training days and the numbers of crossing platform during 10 min probe test. The escape latency of the rHMGB1-treated rats was significantly longer during training on the Morris water maze; the number of platform crosses was also decreased than controls (3.588 ± 0.4206 versus 2.412 ± 0.3328, *P* = 0.0356). Data are presented as mean ± SEM (*n* = 20 rats from each group). rHMGB1, rHMGB1-treated.

Open-field tests showed that the rHMGB1-treated rats spent less time in the center than the control rats (Figure 10A, *n* = 20, *P* = 0.0133, unpaired *t*-test). The rHMGB1-treated rats also had a decrease in the total distance traveled compared with that of the control rats (Figure 10B, *n* = 20, *P* = 0.0108 unpaired *t*-test). The rHMGB1-treated rats spent more time examining novel objects than the control rats (Figure 10C, *n* = 20, *p* = 0.0002). Moreover, the time spent examining the familiar object in object recognition tests was also increased in the rHMGB1-treated rats than the control rats (Figure 10D, *n* = 20, *P* = 0.0107). The escape latency of the rHMGB1-treated rats was significantly longer than that of the control rats during training on the Morris water maze (Figure 10E); the number of platform crosses was also decreased (Figure 10F, *P* = 0.0356). These results indicate that the rHMGB1-treated rats were less exploratory and even had cognitive deficits.

## Discussion

The present study showed that ventricular injection of 0.2 μg rHMGB1 on gestational day 14.5 resulted in epileptogenic CD, which was characterized by cortical disorganization and heterotopia, linearly aligned neurons and deformed neurons, neuroinflammation and hyper neuronal excitability. The rHMGB1 antagonist dexmedetomidine alleviated the neuroinflammation induced by rHMGB1. In addition, rHMGB1-treated rats were more susceptible to seizures after pilocarpine administration than controls after 3 months postnatal and displayed spontaneous SWDs after 5 months postnatal. The SWDs were mainly distributed at approximately 8 Hz and propagated through various cerebral cortices. Further, behavioral tests demonstrated that 0.2 μg rHMGB1-treated rats were less exploratory and even had cognitive deficits. These results suggest that rHMGB1-induced CD preceded epileptogenesis and mimicked the pathological manifestation of human epileptogenic CD.

### The Characteristics of CD Models

The proper development and organization of the brain require normal proliferation, migration, and differentiation of cortical neural progenitors. It is unclear whether neuroinflammation could lead to CD by disrupting these processes. In humans, CD is characterized by dyslamination and disorganized cortical architecture, including dysmorphic neurons and balloon cells (Severino et al., 2020). However, animal models only mimic some CD characteristics, including laminar disorganization, microgyria, focal heterotopia, periventricular heterotopia, and occasional cytomegalic neurons (Luhmann, 2016). Our results show that ventricular injection of 0.2 μg rHMGB1 in rat pups induced cortical dyslamination, heterotopia, microgyria, and even linearly aligned neurons and deformed neurons in the cortex, which are characteristics of CD. Neuronal numbers were decreased in cortical layers V and VI, deformed neurons displayed large soma, increased neurofilament expression in apical dendrites and mildly thickened dendrites, and astrogliosis was also observed in cortical layers I and V. The above phenotypes are features of epileptic CD and promote epileptogenesis (Kielbinski et al., 2016). Astrogliosis is also associated with injury-induced inflammation and precedes the onset seizure of FCD and TSC (Sosunov et al., 2008; Talos et al., 2008; Zeng et al., 2011). These results demonstrate that HMGB1 could be the important factor involved in the development of CD. Previous studies examined the impact of inflammation were induce by lipopolysaccharide (LPS) on the brain of embryonic rodents; the method of LPS injection included the intraperitoneal, tail intravenous, and intra-amniotic administration, but no focal cortical dysplasia (FCD) was observed. It speculates that the inflammation induced by LPS maybe destroy the cortex; LPS and exogenous factors may not enter the fetal brain due to the placental barrier and blood-brain barrier easily. Then, we injected the rHMGB1 into the embryonic brain. The TLR4 and NF-κB were significant increased after rHMGB1 administration. Moreover, rHMGB1-indued changes were partly reversed by dexmedetomidine. This suggested HMGB1 inhibition may be a measure that protects the impaired embryonic brain from the disorder of cortical development.

### The Feature of Various CD Models

To detect the mechanisms of CD and CD-associated epilepsy, CD animal models are essential and often experimentally induced by physical, chemical, or genetic manipulations to the developing brain (Luhmann, 2016). The *in utero* frozen model mimics microgyria, focal heterotopia, and spontaneous epileptic discharges of the hippocampus (Kamada et al., 2013); the prenatal irradiation model reproduces microgyria and heterotopia but does not produce spontaneous epileptic discharges; the prenatal intraperitoneal injection of methylazoxymethanol (MAM) model exhibits cortical disorganization and periventricular nodules. Carmustine 1-3-bis-chloroethyl-nitrosourea (BCNU) was injected intraperitoneally into pregnant rats to mimic laminar disorganization, heterotopias, and cytomegalic neurons, but spontaneous epileptic discharges were not observed in either model (Luhmann, 2016). Moreover, the specific gene was manipulated to mimic CD with epilepsy that was caused by a single genetic abnormality (Lim et al., 2017; Ribierre et al., 2018). Our results included cortical disorganization, microgyria, focal heterotopia, and cortical and periventricular heterotopia; some of these were the characteristics of various models and were also features of human CD (Najm et al., 2018). Moreover, linearly aligned neurons and deformed neurons were also obvious in our model rats; most importantly, spontaneous epileptic discharges from the cortex were also detected. Interestingly, the rHMGB1-treated rats had no extensive pathological changes that were provoked by genetic or general systemic intervention, unlike the TSC knockout, MAM injection, and irradiation models. These results suggest that HMGB1 could promote the development of focal CD.

### The Abnormal Excitation of CD Models

In epileptic CD, dysmorphic neurons are hyperexcitable and could be the pacemaker focus of seizures (Chassoux et al., 2000; Cepeda et al., 2020). In human resected tissues, the N1R immunoreactivity of dysplastic and dysmorphic neurons was increased, and NR2A and NR2B were also overexpressed, which is a feature of hyperexcitable developing neurons (Chassoux et al., 2000; Finardi et al., 2013). In CD animal models, NR2B and NR1 were also upregulated in lesions (Kamada et al., 2013; Deshmukh et al., 2018). Furthermore, *in vitro* ifenprodil reduced the excitability of CD brain tissue slices, and *in vivo* administration of ifenprodil blocked NR2B; these results clarified the important roles of NR2B and NR1 upregulation in increasing epileptogenicity in the frozen rat model of CD (Takase et al., 2008; Wang et al., 2017). In this study, NR1, NR2B, and NR2A were upregulated in cortical lesions, suggesting that excitation was increased and susceptible to seizures.

Studies have shown that GABA neurons are reduced in CD, but GABA terminals are increased surrounding dysplastic neurons, and GABA is an excitatory rather than inhibitory neurotransmitter that enhances neuronal synchronization, leading to seizures (Abdijadid et al., 2015). In this study, GAD65/67-positive neurons displayed normal cell density in cortical lesions. EAAT1 and EAAT2 were also downregulated in cortical lesions. EAAT1 and EAAT2 are the major transporters involved in the uptake of matrix glutamate (Bjørnsen et al., 2014). Therefore, the downregulation of EAAT1 and EAAT2 increased matrix glutamate, resulting in the neuronal hyperactivity, which could be the mechanism of epileptic discharge in CD. In summary, our results indicate that HMGB1 promoted the development of abnormal excitable CD, which increased susceptibility to seizures.

### Spike-Wave Discharges in CD

Previous studies have shown that spike-wave discharges (SWDs) appear to be in remission before adolescence (Sun et al., 2016; Williams et al., 2016). However, in our study, SWDs were recorded in adult rats, which suggested that the brains of rHMGB1-treated rats could be immature. In the frozen model of CD, SWDs were also detected at a higher frequency with longer durations during slow-wave sleep (Williams et al., 2016). The SWD pattern was similar to that of spontaneous SWDs that have been observed in absence epilepsy and similar to the spike-wave that occurs in human absence seizures and electrical status epilepticus (Coenen and Van Luijtelaar, 2003; Sun et al., 2016; Antwi et al., 2019). However, it is still unclear whether the mechanism of epileptogenesis is associated with SWDs. Some studies have suggested that evoked epileptiform activities can propagate to almost all cortical areas and could be involved in thalamocortical dysfunction and excitation and inhibition imbalance (Luhmann et al., 1998). Therefore, some hypotheses have suggested that spike waves and spindles share similar circuit mechanisms (Steriade, 2006; Beenhakker and Huguenard, 2009). In this study, the EEG displayed a pattern of epileptiform discharges that closely resembled spike waves. A total of 8/10 adult rats exhibited multiple spontaneous non-convulsive seizure epochs during 24-h intracranial EEG monitoring that were composed of bursts of rhythmic high-amplitude spike waves, and the frequency ranged from 6 to 10 Hz (theta-alpha band). In contrast, the controls did not display epileptic discharges and seizures (0/10). Therefore, theta and alpha band rhythms may play an important role in the formation of spike-wave epileptic discharges. Next, the degree of synchrony was compared among the different brain regions through coherence and cross-correlation analyses. Intriguingly, the results revealed that SWDs between the frontal and occipital regions were highly synchronous (distance 10.5 mm); in particular, ictal spikes were concentrated to approximately 8 Hz, suggesting that SWDs were highly generalized and that these low frequencies may be involved in generalization and synchronization in different remote brain regions.

### The Mechanism of HMGB1-Evoked CD

High-mobility group box 1 on the neuronal surface and in the extracellular matrix promotes neurite outgrowth, cell migration, and neuroinflammation. In the E14.5 embryonic rodent brain, HMGB1 is widely present throughout the developing cortex and thalamic region, and HMGB1 is restricted to the newly generated neurons of the cortical plate (Guazzi et al., 2003; Fang et al., 2012). At E16, HMGB1-positive neurons were observed moving to the cortex from the subventricular and ventricular zones (Guazzi et al., 2003). However, beginning at E18, the expression level of HMGB1 was downregulated to the level observed in adults, in which HMGB1 was only present in regions of continuous neurogenesis, such as the hippocampus (Guazzi et al., 2003). HMGB1 has been shown to trigger hippocampal neurogenesis after brain injury (Meneghini et al., 2010). Moreover, HMGB1 knockdown induced a smaller head size in zebrafish larvae, which reduced the number of proliferating cells in the neurogenesis zones and the number of neurons in the cortex (Zhao et al., 2011). Furthermore, HMGB1 injection partially rescued the above phenotype. In addition, cultured cerebellar granule neurons on postnatal days 4 to 7 displayed more extensive neurites and migrated out of the core of cultures in media containing HMGB1. Blocking HMGB1 reduced N18 neuroblastoma and C6 glioma cell migration in a dose-dependent manner (Fang et al., 2012). Therefore, in this study, we chose to inject rHMGB1 into the E14.5 embryonic rat brain. The results demonstrated that rHMGB1 treatment decreased the number of neurons and promoted cortical neuron disorganization to form focal cortical lesions, microgyria, cortical and periventricular heterotopia, and subcortical heterotopia. Some studies have shown that disorganized superficial cortical layers are a representative feature of FCD Ia, and subcortical heterotopia is a class of CD (Palmini et al., 2004; Barkovich et al., 2005). Further, the anti-inflammatory compound dexmedetomidine alleviated the cortical changes induced by rHMGB1. Therefore, our results indicate that HMGB1 could induce epileptic CD through neuroinflammation in a manner similar to human CD.

### Behavioral Abnormalities

Brain developmental delay and cognitive impairment are common comorbidities of intractable epilepsy-associated CD. A previous rat model of CD demonstrated impaired spatial memory, which depends on hippocampal subregions (CA1, CA3, and DG) (Clark et al., 2007). It is known that recognition memory relies mostly on cortical areas, whereas some studies have suggested that the hippocampus is not essential for object recognition (Zhou et al., 2011). Behavioral deficits were observed in the rHMGB1-treated rats, similar to reports of the chemical and physical models of CD. The rHMGB1-treated rats were less exploratory, spent more time on the novel and familiar objects, and had a longer escape latency than the control rats. Thus, our results indicate that rHMGB1 treatment could broadly affect the whole brain network to result in cognitive impairment.

### Endogenous HMGB1 Is the Key Factor of CD Development

During pregnancy, environmental changes during brain development can result in alterations in the cortical organization, such as altered brain size and aberrant neuronal clusters. To date, animal models of CD have mainly depended on physical and chemical damage. Previous studies have demonstrated that chemical and physical insults in the external environment (e.g., MAM and frozen models) resulted in the impairment of neurogenesis, the disruption of cell death and apoptosis, the migration of pyramidal neurons, and the tangential migration of interneurons (Luhmann, 2016). The above resulting malformations were similar to those observed in resected tissues from patients with CD. However, those treatments were exogenous and dependent on the time and extent of impairment in the developing brain rather than on endogenous causes. Furthermore, various insults can induce CD in the pre-or perinatal period, such as hypoxia-ischemia and viral infection, which all promote HMGB1 release to amplify neuroinflammation and disrupt cortical development (Blümcke et al., 2011). In conclusion, HMGB1 is the endogenous intersection point of various impairments resulting from chemical, physical and genetic manipulations in the rodent model of CD. Therefore, the rHMGB1-treated rats partly simulated the pathological features of clinical CD resulting from endogenous damaging factors.

## Conclusion

Our results show that ventricular injection of 0.2 μg rHMGB1 on E14.5 could contribute to the process of epileptic CD development, indicating that increased endogenous HMGB1 levels resulting from various reasons during pregnancy could induce epileptogenic CD.

## Data Availability Statement

The original contributions presented in the study are included in the article/Supplementary Material, further inquiries can be directed to the corresponding author.

## Ethics Statement

The animal study was reviewed and approved by Animal Experiments and the Internal Animal Care and Use Committee of the Army Medical University.

## Author Contributions

XY and XZ contributed equally to this study. SYL, HY, and XY conceived and designed the research. SYL, CZ, and SQL supervised the experiments. XY, XZ, ZW, GZ, YM, KH, and TW performed the Nissl staining, WB, and IHC. XZ, XY, and KS monitored the EEGs. XY and GL carried out the EEG analysis and statistical analysis. XY wrote the first draft of the manuscript and revised it with the suggestions of the other authors. SYL reviewed the manuscript and provided advice. All authors have approved the submission of the final manuscript.

## Conflict of Interest

The authors declare that the research was conducted in the absence of any commercial or financial relationships that could be construed as a potential conflict of interest.

## Supplementary Material

Supplementary Figure 1 Schematic diagram illustrating the experimental design and the number of rats used in the different experimental procedures. IHC, immunohistochemistry; IF, immunofluorescence; WB, western blot; EEG, electroencephalogram; SE, status epilepticus.

Supplementary Figure 2 Morphological effects of rHMGB1 on cortex. **(A–E)** The distribution of neuronal sizes in layer II–VI. The proportion of big size neurons (180–240 μm^2^) were increased in cortical layer II and III **(A,B)**, meanwhile the proportion of small size neurons (60–80 μm^2^) were increased in cortical layer IV, V and VI of 0.2 μg rHMGB1-treated rats versus controls **(C–E)**. **(F–J)** The neuronal shape in cortical layer II–VI. There were significant difference shape of neurons in cortical layer II–VI of 0.2 μg rHMGB1-treated rats versus controls (^****^*P* < 0.0001, ^∗∗^*P* = 0.0043, ^****^*P* < 0.0001, ^****^*P* < 0.0001, respectively). **(K–O)** The neuronal orientation in cortical layer II–VI. Only layer IV and V displayed the abnormal neuronal orientation in 0.2 μg rHMGB1-treated rats versus controls (**M,N**, ^∗∗^*P* = 0.0024 and ^∗∗∗^*P* < 0.0001, respectively). However, we did not observe the evident abnormal of neuronal sizes, shape and orientation in cortical layer II–VI of 0.1 μg rHMGB1-treated rats compared with controls. Data are represented as mean ± SEM (*n* = 6 rats from each group). rHMGB1, rHMGB1-treated. L2–L6; cortical layer II–VI.

Supplementary Figure 3 Immunohistochemical evaluation of the cortical lesions. **(A–I)** NR1, NR2A and NR2B immunoreactivity was significantly increased in cortical lesions (0.25 ± 0.01 versus 0.32 ± 0.02 ^∗∗^*P* = 0.0104; 0.24 ± 0.01 versus 0.29 ± 0.01 ^∗^*P* = 0.0253; 0.18 ± 0.02 versus 0.32 ± 0.01 ^****^*P* < 0.0001; respectively). **(J–L)** The immunoreactivity of GAD65/67 was not different between controls and cortical lesions (0.28 ± 0.03 versus 0.25 ± 0.02 *P* = 0.40). **(M–R)** EAAT1 and EAAT2 was decreased in cortical lesions versus controls (0.29 ± 0.01 versus 0.22 ± 0.01 ^∗∗^*P* = 0.0017, 0.31 ± 0.02 versus 0.26 ± 0.01 ^∗^*P* = 0.0397; respectively). Data are represented as mean ± SEM (*n* = 5 rats from each group). rHMGB1, rHMGB1-treated; NR2A, *N*-methyl-D-aspartate receptor 2A; NR2B, *N*-methyl-D-aspartate receptor 2B; NR1, *N*-methyl-D-aspartate receptor 1; GAD65/67, glutamic acid decarboxylase 65/67; EAAT1, excitatory amino acid transporter 1; EAAT2, excitatory amino acid transporter 1. Scale bars, 50 μm.

Supplementary Figure 4 The immunohistochemical examination of the hippo- campus. **(A–C)** There were stronger immunoreactivity of NR2A in CA3 of rHMG B1-treated rats versus controls (^∗^*P* = 0.0138); but there was no difference in other subregion of HMGB1 rat compared to controls. **(D–F)** Immunoreactivity of NR2B were also stronger in CA3 of rHMGB1-treated rats compared with control group (^∗^*P* = 0.0149), there was also no difference in other subregion of rHMGB1-treated rats compared to controls. Data are represented as mean ± SEM (*n* = 5 rats from each group). rHMGB1, rHMGB1-treated. Scale bars, 50 μm.
