# Supplementary material for: Abnormal Rat Cortical Development Induced by Ventricular Injection of rHMGB1 Mimics the Pathophysiology of Human Cortical Dysplasia
Source: Front Cell Dev Biol. 2021 Mar 4;9:634405. doi: 10.3389/fcell.2021.634405 (PMC7969805; doi:10.3389/fcell.2021.634405)
Supplement: Supplementary Figure 1 — Schematic diagram illustrating the experimental design and the number of rats used in the different experimental procedures. IHC, immunohistochemistry; IF, immunofluorescence; WB, western blot; EEG, electroencephalogram; SE, status epilepticus. [file Presentation_1.pdf]

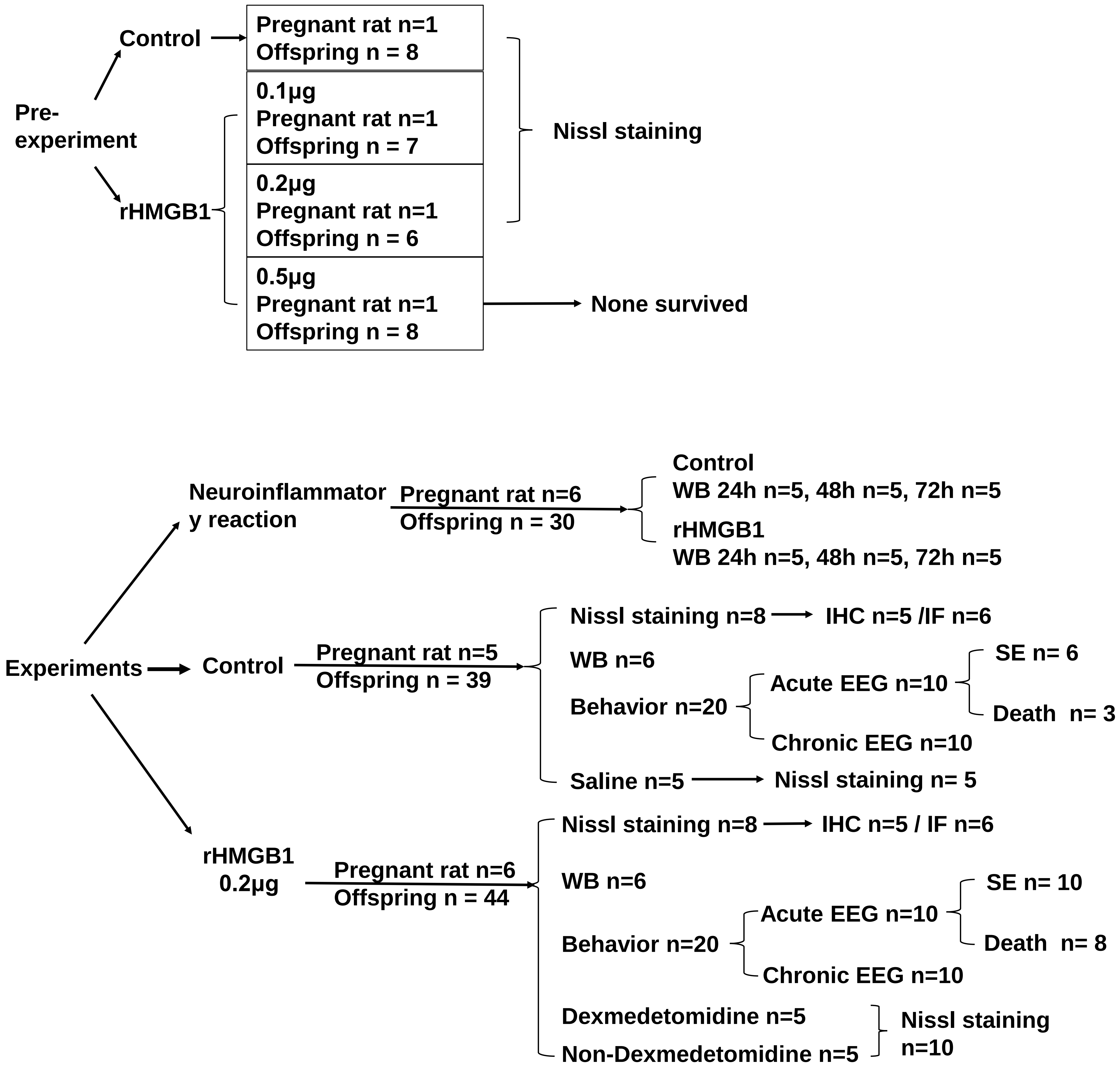

Supplementary Figure 1 | Schematic diagram illustrating the experimental design and the number of rats used in the different experimental procedures. IHC, immunohistochemistry; IF, immunofluorescence; WB, western blot; EEG, electroencephalogram; SE, status epilepticus.

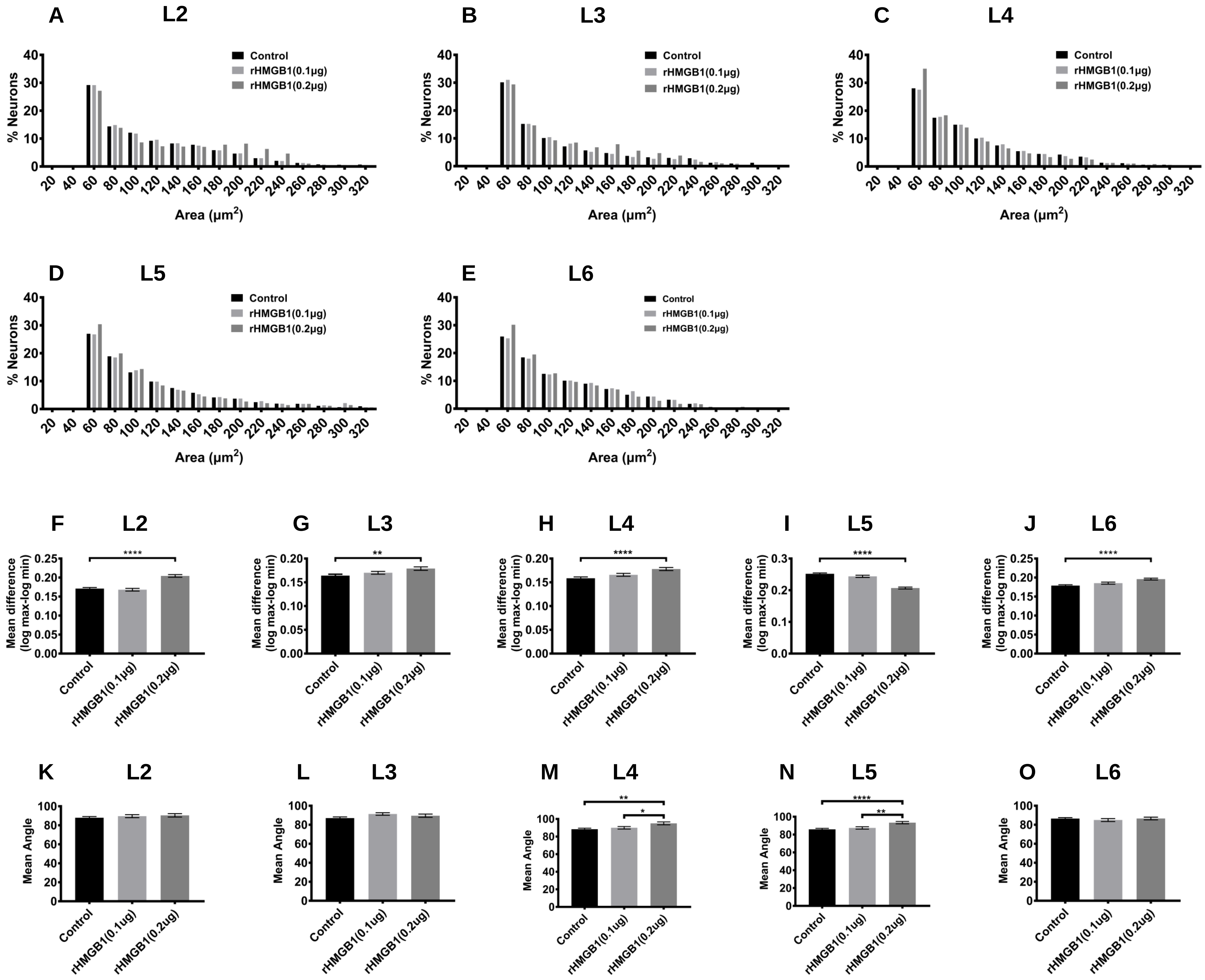

Supplementary Figure 2 | Morphological effects of rHMGB1 on cortex. (A–E) The distribution of neuronal sizes in layer II–VI. The proportion of big size neurons (180–240 μm^2) were increased in cortical layer II and III (A,B), meanwhile the proportion of small size neurons (60–80 μm^2) were increased in cortical layer IV, V and VI of 0.2 mg rHMGB1-treated rats versus controls (C–E). (F–J) The neuronal shape in cortical layer II–VI. There were significant difference shape of neurons in cortical layer II–VI of 0.2 μg rHMGB1-treated rats versus controls (*****P* < 0.0001, ***P* = 0.0043, *****P* < 0.0001, *****P* < 0.0001, respectively). (K–O) The neuronal orientation in cortical layer II–VI. Only layer IV and V displayed the abnormal neuronal orientation in 0.2 μg rHMGB1-treated rats versus controls (M,N, ***P* = 0.0024 and *****P* < 0.0001, respectively). However, we did not observe the evident abnormal of neuronal sizes, shape and orientation in cortical layer II–VI of 0.1 μg rHMGB1-treated rats compared with controls. Data are represented as mean ± SEM (n = 6 rats from each group). rHMGB1, rHMGB1-treated. L2–L6; cortical layer II–VI.

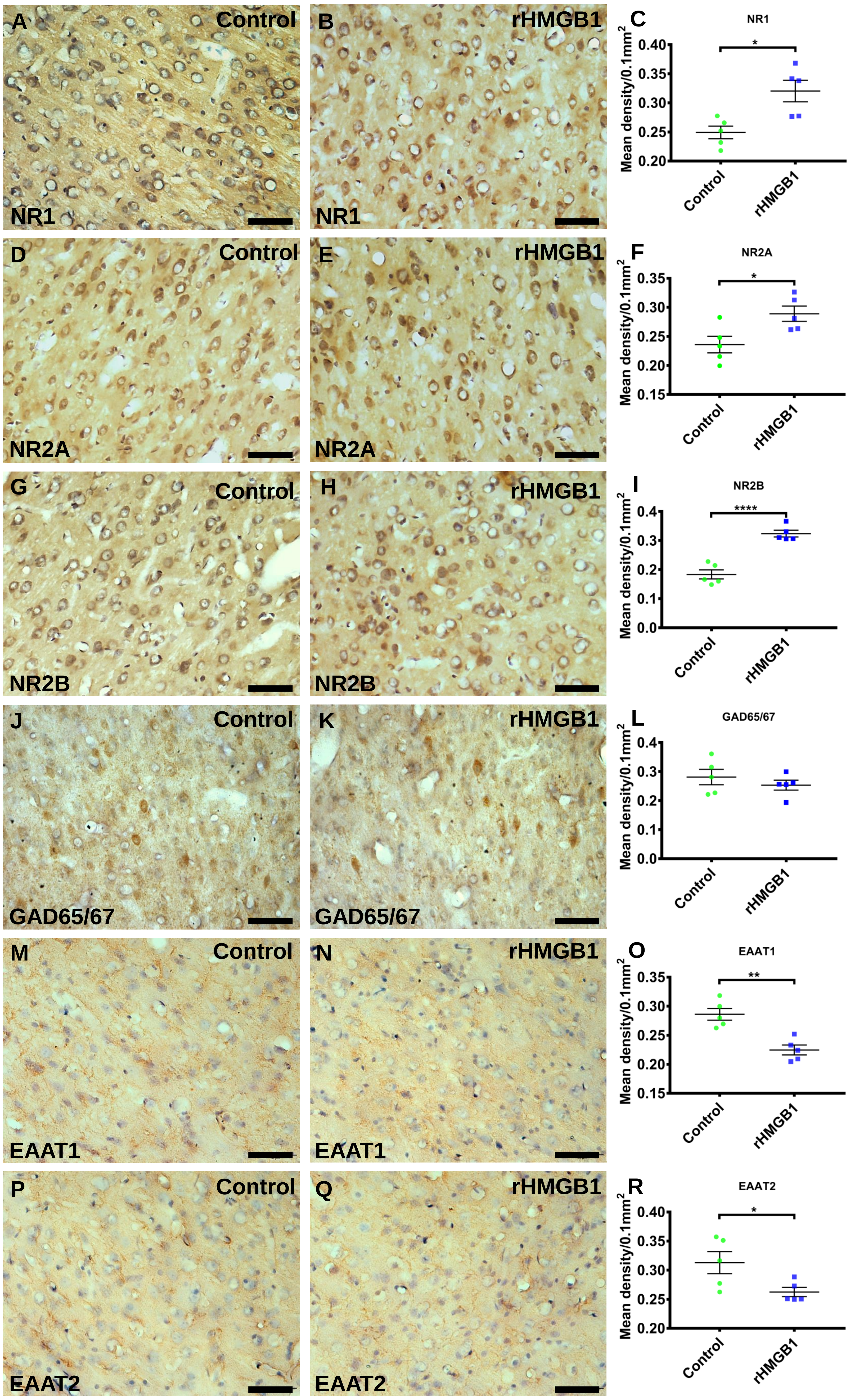

Supplementary Figure 3 | Immunohistochemical evaluation of the cortical lesions. (A–I) NR1, NR2A and NR2B immunoreactivity was significantly increased in cortical lesions (0.25±0.01 versus 0.32±0.02 ***P* = 0.0104; 0.24±0.01 versus 0.29±0.01 **P* = 0.0253; 0.18±0.02 versus 0.32±0.01 *****P* < 0.0001; respectively). (J–L) The immunoreactivity of GAD65/67 was not different between controls and cortical lesions (0.28±0.03 versus 0.25±0.02 *P* = 0.40). (M–R) EAAT1 and EAAT2 was decreased in cortical lesions versus controls (0.29±0.01 versus 0.22±0.01 ***P* = 0.0017, 0.31±0.02 versus 0.26±0.01 **P* = 0.0397; respectively). Data are represented as mean ± SEM (n = 5 rats from each group). rHMGB1, rHMGB1-treated; NR2A, N-methyl-D-aspartate receptor 2A; NR2B, N-methyl-D-aspartate receptor 2B; NR1, N-methyl-D-aspartate receptor 1; GAD65/67, glutamic acid decarboxylase 65/67; EAAT1, excitatory amino acid transporter 1; EAAT2, excitatory amino acid transporter 1. Scale bars, 50 mm.

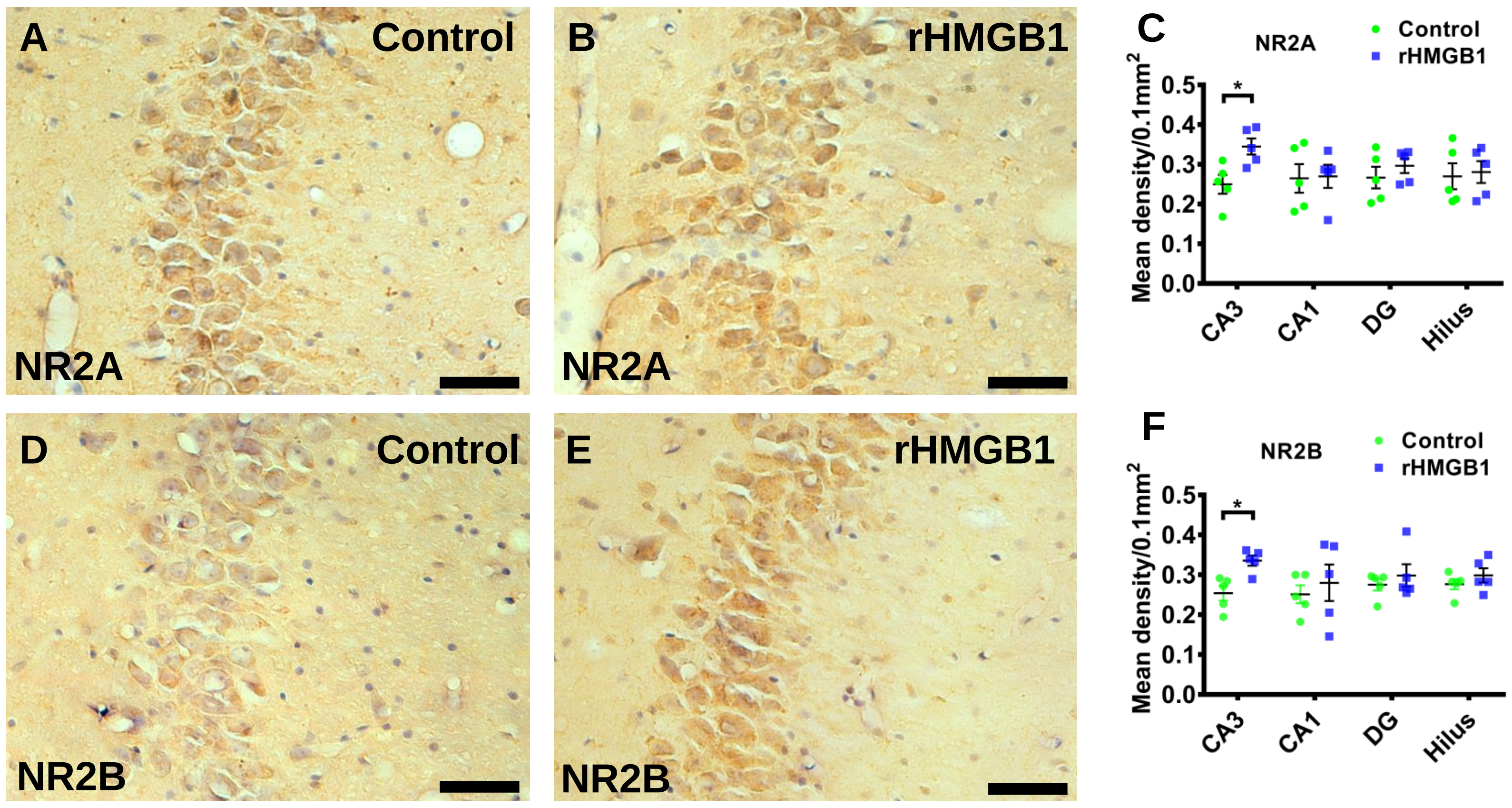

Supplementary Figure 4 | The immunohistochemical examination of the hippocampus. (A–C) There were stronger immunoreactivity of NR2A in CA3 of rHMGB1-treated rats versus controls (*P = 0.0138); but there was no difference in other subregion of HMGB1 rat compared to controls. (D–F) Immunoreactivity of NR2B were also stronger in CA3 of rHMGB1-treated rats compared with control group (*P = 0.0149), there was also no difference in other subregion of rHMGB1-treated rats compared to controls. Data are represented as mean ± SEM (n = 5 rats from each group). rHMGB1, rHMGB1-treated; NR2A, N-methyl-D-aspartate receptor 2A; NR2B, N-methyl-D-aspartate receptor 2B. Scale bars, 50 mm.
